# Comparison of Host Gene Expression Profiles in Spleen Tissues of Genetically Susceptible and Resistant Mice during ECTV Infection

**DOI:** 10.1155/2017/6456180

**Published:** 2017-12-21

**Authors:** Wen-Yu Cheng, Huai-Jie Jia, Xiao-Bing He, Guo-Hua Chen, Yuan Feng, Chun-Yan Wang, Xiao-Xia Wang, Zhi-Zhong Jing

**Affiliations:** ^1^State Key Laboratory of Veterinary Etiological Biology, Key Laboratory of Veterinary Public Health of Agriculture Ministry, Lanzhou Veterinary Research Institute, Chinese Academy of Agricultural Sciences, Lanzhou, Gansu 730046, China; ^2^School of Public Health, Lanzhou University, Lanzhou 730000, China

## Abstract

Ectromelia virus (ECTV), the causative agent of mousepox, has emerged as a valuable model for investigating the host-*Orthopoxvirus* relationship as it relates to pathogenesis and the immune response. ECTV is a mouse-specific virus and causes high mortality in susceptible mice strains, including BALB/c and C3H, whereas C57BL/6 and 129 strains are resistant to the disease. To understand the host genetic factors in different mouse strains during the ECTV infection, we carried out a microarray analysis of spleen tissues derived from BALB/c and C57BL/6 mice, respectively, at 3 and 10 days after ECTV infection. Differential Expression of Genes (DEGs) analyses revealed distinct differences in the gene profiles of susceptible and resistant mice. The susceptible BALB/c mice generated more DEGs than the resistant C57BL/6 mice. Additionally, gene ontology and KEGG pathway analysis showed the DEGs of susceptible mice were involved in innate immunity, apoptosis, metabolism, and cancer-related pathways, while the DEGs of resistant mice were largely involved in MAPK signaling and leukocyte transendothelial migration. Furthermore, the BALB/c mice showed a strong induction of interferon-induced genes, which, however, were weaker in the C57BL/6 mice. Collectively, the differential transcriptome profiles of susceptible and resistant mouse strains with ECTV infection will be crucial for further uncovering the molecular mechanisms of the host-*Orthopoxvirus* interaction.

## 1. Introduction

Poxviruses comprise a diverse family of double-stranded DNA viruses that remain a threat to the human and livestock, despite the fact that naturally circulating variola virus (VARV), the causative agent of smallpox, was eradicated decades ago [1–3]. The possibility that clandestine stocks are being held by rogue nations or terrorist groups, as well as an increase in the frequency of zoonotic poxvirus infections, including monkeypox virus (MPXV), has increased attention in recent years [3–7]. VARV has a restricted host range and is known to only infect humans. Closely related* Orthopoxviruses* such as ectromelia virus (ECTV) are the best surrogate for the study of VARV in small animal models, as it also has a restricted host range and, in mice, the resulting disease shares common features with VARV [7–10].

ECTV has a very narrow host range and infection in mice causes mousepox. All laboratory mouse strains can be infected with very low doses of infectious particles, but different mouse genotypes display different susceptibility to lethal infection with ECTV [9–11]. Strains such as BALB/c, DBA/2, DBA2/J, CBA/H, and A/J are considered susceptible to severe disease, while C57BL/6, C57BL/10, AKR, and 129 mice show very low morbidity and mortality and limited pathology and are classified as resistant [12–15]. In addition to virus strain and other factors, such as route of infection, age, sex, and immune status, host genetic background is a critical factor which governs resistance to mousepox [9, 11]. At present, at least four known genetic loci have been identified in resistant inbred and out-bred mice [9, 16, 17].* Ly49H* (also called* resistance to mousepox-1*,* Rmp-1*) maps to the natural killer gene complex (NKC) and activates NK cells to control early virus replication in C57BL/6 mice, but this is lacking in BALB/c mice [11, 18–20]. Other loci, such as the* Rmp-2* locus that maps near the complement component C5 gene,* Rmp-3* locus that is linked to the MHC and is also gonad-dependent, and the* Rmp-4* locus that maps near the selectin gene complex, are also responsible for resistance to ECTV infection [11, 16, 21]. In addition, the humoral and cell mediated immune responses to ECTV infection are very different between BALB/c and C57BL/6 mice [11, 22–26]. C57BL/6 mice can generate robust NK cell, cytotoxic T lymphocytes (CTLs), and IFN-*γ* responses. However, these responses are suboptimal but high levels of IL-4 are produced in BALB/c mice [11, 12, 26–28]. A polarized type 1 cytokine response, in particular IFN-*γ*, and a potent cell mediated immune response determine the genetic resistance of C57BL/6 mice to mousepox. In contrast, a polarized type 2 cytokine response is generated in susceptible mouse strains (BALB/c and A/J), which is associated with a weak or absent CTL response, resulting in uncontrolled virus replication and animal death [11, 12]. Additional factors involved in innate and adaptive immunity are also required for inherent resistance to mousepox [11]. Type I IFNs induced by viral proteins and nucleic acids through the recognition of pathogen recognition receptors (PRRs) are essential for inherent resistance to mousepox in C57BL/6 mice [28–30]. Deficiencies in TLR9-MyD88-IRF7 and STING-IRF7/NF-*κ*B result in inefficient production of type I IFNs, higher mortality rates, and accelerated death in C57BL/6 mice [28]. Other components of innate immunity, such as phagocytes, antigen-presenting cells, granzymes A and B, nitric oxide synthase 2, IL-12, and IL-18, also play essential roles in inherent resistance to mousepox [31–37].

Innate immunity is required, but not sufficient, for inherent resistance to mousepox. Decades of work on adaptive immunity have shown that a number of factors, at the molecular and cellular level, are essential to control ECTV infection in resistant strains [11]. B lymphocytes produce antibodies that can directly neutralize virus particles to prevent infection, and cluster of differentiation 40 (CD40) is essential for efficient antibody production and isotype switching [11, 22]. T cells are also involved in conveying resistance to primary ECTV infection [11]. CD8^+^ T cell responses to ECTV in C57BL/6 mice are extremely strong and bond with the cell surface major histocompatibility complex I (MHC I), which recognizes viral peptides, resulting in high susceptibility to mousepox [11, 22]. The function of CD4^+^ T cells in resistance to mousepox is to produce anti-ECTV antibodies and kill the infected cells in a perforin-dependent manner [24, 38].

Despite the fact that decades of work have contributed to our knowledge of the pathogenesis and immunobiology of ECTV infection* in vivo*, the systemic differences between susceptible and resistant mouse strains during the ECTV infection have not yet been investigated. On the other hand, microarray methodology has been developed as a high throughput method to simultaneously analyze large datasets of gene expression patterns under various biological conditions [39]. Thus, to obtain a comprehensive view of the host responses to ECTV infection in different mouse strains at the mRNA level, we performed cDNA microarray analysis of mRNAs obtained from the spleens of two mouse strains, one susceptible and another one resistant to ECTV. This analysis revealed the shared and distinct expression profiles and strain-specific pathogenesis may be due to the differentially active pathways and differences of gene expression levels in the two different mouse strains.

## 2. Materials and Methods

### 2.1. Ethics Statement

All animal work was conducted according to the Good Animal Practice Requirements of the Animal Ethics Procedures and Guidelines of the People's Republic of China. All experimental protocols were approved by the Animal Ethics Committee of Lanzhou Veterinary Research Institute, Chinese Academy of Agricultural Science (permit number LVRIAEC2016-008).

### 2.2. Mice and Virus

Seven- to nine-week-old BALB/c and C57BL/6 SPF mice were purchased from the Experimental Animal Center of Lanzhou University, China. Upon arrival, animals were housed in a biosafety level 3 room and given free access to commercial mouse chow and water. After a one-week acclimatization period, the two strains of mice were randomly assigned to three experimental groups, with 5 mice per group.

The wild-type strain of ECTV was originally isolated from naturally infected laboratory mice and then propagated in Vero cells (unpublished data). Virus was confirmed by PCR using the specific primers (5′-ATGGACGGAACTCTTTTC-3′ and 5′-AACTTCATCGTTGCGTTTAC-3′) and sequenced. The experimental infection of susceptible BALB/c mice was less virulent than the ECTV-Moscow strain (data unpublished). Plaque-purified ECTV was serially passaged in Vero cell for 21 generations, and virus titer was measured using a 50% tissue culture infective dose (TCID_50_) assay.

### 2.3. Virus Infection and Confirmation of Infection

Infection groups comprising 10 BALB/c and C57BL/6 mice were anesthetized and infected subcutaneously into the abdomen with 100 *µ*L PBS containing 10^4^ TCID_50_ of ECTV. Ten uninfected age-matched mice (5 BALB/c and 5 C57BL/6) served as the control group and were euthanized by cervical dislocation before the spleen tissues were isolated. On days 3 and 10 after infection, 5 mice from each infection group were sacrificed and whole spleen tissues were harvested. All spleen tissues from each group were pooled into a cell culture plate and cut into pieces using surgical scissors. A total of 1.6 grams pooled spleen tissues were equally divided into 4 tubes, then snap frozen in liquid nitrogen, and stored at −70°C. For confirmation of infection, genomic DNA was extracted from the spleen tissues of infected or control groups. Then a PCR was performed to detect infection using the above-mentioned primers.

### 2.4. Virus Titration

To determine virus titers, 0.25 mL PBS was added to 0.25 g pooled spleen tissues and the tissue was homogenized using a disposable tissue-grinding pestle (Sangon, Shanghai, China). The homogenized samples were frozen and thawed three times. Virus titration was assessed using a TCID_50_ assay. Briefly, 100 *μ*L Vero cell suspension, containing 2  ×  10^4^ cells in DMEM with 10% fetal bovine serum (FBS) (Invitrogen, Carlsbad, CA, USA), was seeded into each well of a 96-well plate and incubated at 37°C under 5% CO_2_. After 24 h incubation, 25 *μ*L of 10-fold serial dilutions was added to each well, with 8 replicates per dilution. Plates were incubated at 37°C for 10 days and checked daily for characteristic cytopathic effect (CPE). TCID_50_ end-point titers were calculated using the Reed and Muench method [40].

### 2.5. Histopathology

Spleens were harvested and fixed with 10% neutral buffered formalin solution at 4°C for 4 h and then were embedded in paraffin. The paraffin-embedded specimens were cut into 5 *μ*m thick sections and stained with hematoxylin-eosin (H&E). Each slide of the samples was photographed with a digital optical microscope (Olympus, Tokyo, Japan).

### 2.6. Microarray Analysis

Frozen samples were sent to CapitalBio Co. (Beijing, China), who performed the microarray experiment according to protocols provided by Affymetrix. Briefly, total RNA was isolated from the samples and the quality and quantity of RNA were assessed using formaldehyde agarose gel electrophoresis and spectrophotometry. Biotin-labeled fragmented cRNA samples were subjected to hybridization with GeneChip Mouse Genome Arrays (GeneChip® Mouse Genome 430 2.0) (Affymetrix, Santa Clara, California, USA), which contained 39000 probes representing 34000 mouse genes. Hybridization was performed at 45°C in an Affymetrix GeneChip Hybridization Oven 640 (Affymetrix, Santa Clara, California, USA), with rotation, for 16 h. Arrays were scanned using a confocal scanner (LuxScan 10K-A, CapitalBio, Beijing, China) and images were analyzed using SpotData software (CapitalBio, Beijing, China). Scanned images were assessed first by visual inspection and then analyzed to generate raw data files that were saved as  .cel files using the default settings of the Gene Chip operating software. All data were deposited into GEO (Gene Expression Omnibus) database at http://www.ncbi.nlm.nih.gov/geo/info/linking.html under the accession number GSE100644. Microarray data were analyzed by using Bio MAS (molecule annotation system) 3.0 software (CapitalBio Corporation, Beijing, China). Using the criterion of cutoff limitation as a fold change ≥ 2 or ≤0.5, differential expression genes were screened and clustered.

### 2.7. Validation of Microarray Data

Nine DEGs from each time point were selected and used to quantify gene expression levels using real-time quantitative PCR. A total of 26 DEGs were verified and expression levels were normalized against the housekeeping standard, glyceraldehyde-3-phosphate dehydrogenase (GAPDH). Primers were designed and synthesized by Sangon Biotech Company, and sequences are listed in Table S1. Total RNA was extracted using TRIzol Reagent (Invitrogen, Carlsbad, CA, USA) and reverse-transcribed to single strand cDNA using a first strand cDNA synthesis kit (AMV, Roche, Germany) according the manufacturer's instructions. Real-time quantitative PCR was performed using SG Fast qPCR Master Mix Kit (BBI, Shanghai, China) in a final volume of 20 *μ*L, containing 10 *μ*L 2x SYBR Green qPCR Master Mix, 4 *μ*M each primer, 7.2 *μ*L nuclease-free water, and 2 *μ*L cDNA. PCR amplification was performed and run in triplicate under the following conditions: one cycle of 95°C for 3 min, followed by 40 cycles of 95°C for 7 s, 57°C for 30 s, and 72°C for 15 s. Gene expression was analyzed using the 2^−ΔΔCt^ method.

## 3. Results

### 3.1. Virus Infection and Viral Loads in the Spleen Tissues from ECTV-Infected Mice

**Figure 1:**
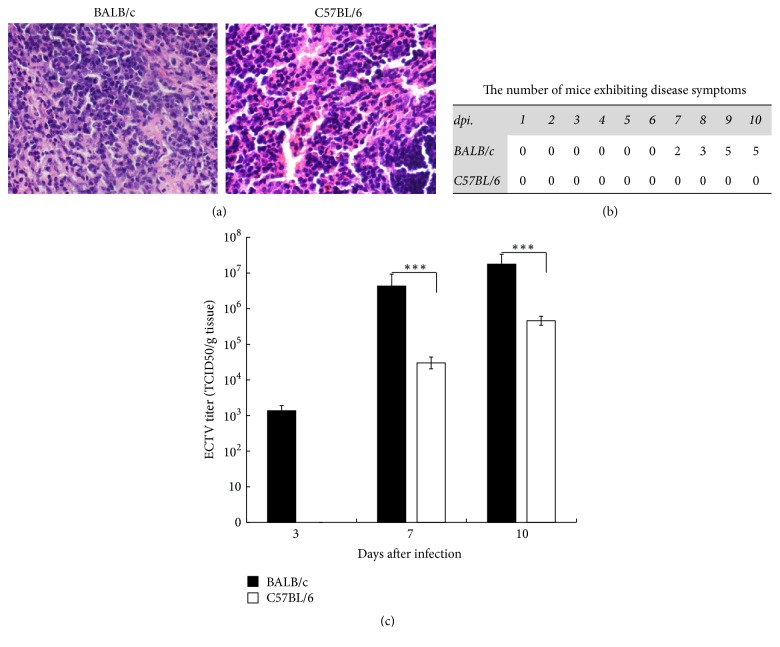
(a) Spleen sections of the indicated mice at 10 dpi stained with H&E at 10 dpi. (b) The number of BALB/c and C57BL/6 mice exhibiting disease symptoms after ECTV infection. Five mice in each group were injected with 10^4^ TCID_50_ of ECTV into the abdominal subcutaneous tissue. The status of infected mice was checked daily. (c) ECTV titers in spleen tissues of susceptible and resistant mice. Groups of BALB/c and C57BL/6 mice infected with ECTV were killed on the days indicated (3, 7, and 10 dpi). Viral titers in pooled spleen of each group were determined with three replications. *∗∗∗* means *P* < 0.001.

Genetically susceptible BALB/c and resistant C57BL/6 mice were injected with 10^4^ TCID_50_ of ECTV (in 100 *µ*L PBS) into the abdominal subcutaneous tissue. After infection, the BALB/c mice began to exhibit disease symptoms at 7 days after infection (dpi) and one animal succumbed to the disease at 10 dpi. The C57BL/6 mice developed no significant symptoms during the course of the experiment and exhibited no mortality (Figure 1(b)). Spleen tissues were chosen because of the essential roles for the induction of protective antiviral immune responses to ECTV and the site for virus replication [11, 28]. Moreover, the viral loads and the level of responses to ECTV in the spleen are discrepant between susceptible BALB/c and resistant C57BL/6 mice [11]. Our preexperiment of pathological sections from ECTV-infected BALB/c and C57BL/6 mice displayed significantly higher pathology in the former mice at 10 dpi (Figure 1(a)). We next measured ECTV viral titers in pooled spleen tissues from the two mouse strains at 3, 7, and 10 dpi. Virus particles were detected in spleen tissues from both mouse strains. In BALB/c mice, a low virus titer was detected as early as 3 dpi and continued to increase with time (Figure 1(c)). In contrast, the virus was not detected in C57BL/6 mice at 3 dpi but reached up to 10^4.48^ and 10^5.66^ TCID_50_/gram tissue by 7 and 10 dpi, respectively (Figure 1(c)). Despite the undetected virus particles at 3 dpi, the presence of virus genomes was confirmed by PCR assay (data not shown). In general, compared to susceptible BALB/c mice, lower viral loads were detected in resistant C57BL/6 mice at all time points, suggesting that ECTV infects efficiently cells of susceptible BALB/c mice.

### 3.2. Changes in the Transcriptome Profile of Spleens from BALB/c and C57BL/6 Mice during ECTV Infection

The overriding aim of these studies is to elucidate host transcriptome profile changes caused by ECTV infection, by comparing results from genetically susceptible and resistant mice. Spleen tissues were isolated from BALB/c and C57BL/6 mice at 3 and 10 dpi and used for microarray analysis. Both profiles were compared to samples from mock-infected control mice. The differentially expressed genes (DEGs) were filtered using the criterion of cutoff limitation as a fold change ≥ 2 or ≤0.5. After normalization, a total of 744 genes were expressed differentially (with 470 up- and 274 downregulated) in BALB/c mice and approximately half of the number of genes (361) were found to be altered (123 up- and 238 downregulated) in C57BL/6 mice at 3 dpi. At 10 dpi, more genes were perturbed in both BALB/c and C57BL/6 mice. Scrutiny of the data showed that 2184 genes (with 1453 up- and 731 downregulated) were altered in susceptible BALB/c mice, while only 1619 DEGs (540 up- and 1079 downregulated) were perturbed by ECTV infection in C57BL/6 mice (Table 1 and Table S2). Of note, more genes were upregulated over the time course in BALB/c mice than they were in C57BL/6 mice.

To further understand the transcriptome profile changes in genetically susceptible and resistant mice during ECTV infection, we listed the genes that were most significantly up- or downregulated (fold change in expression) in the two mice strains at 3 and 10 dpi. As shown in Table 2, different genes were altered in each of the two strains during ECTV infection. At 3 dpi, interferon-stimulated genes (ISGs), including* Gbp1* (guanylate-binding protein 1),* Gbp2, *and* Iigp1 *(interferon inducible GTPase 1), were upregulated in the susceptible mice, whereas only* Ifn-ζ* (interferon zeta) was slightly upregulated in the C57BL/6 mice at this time point. Among the genes that were upregulated at 10 dpi,* GzmB* (granzyme B) showed the greatest fold change in both strains. Other granzymes, such as* GzmD* and* GzmK*, were also upregulated in both strains.* Ifn*-*γ* (interferon gamma) was activated during the later stage of infection, with a 24.2-fold increase in BALB/c mice (BALB/c 10 dpi versus BALB/c uninfected) and a 5.2-fold increase in C57BL/6 mice (C57BL/6 10 dpi versus C57BL/6 uninfected). Interestingly,* Gbp1*,* Gbp2,* and* Iigp1 *which are induced by IFN-*γ* were significantly upregulated at 3 dpi while IFN-*γ* was upregulated late (10 dpi), suggesting that basic expression of IFN-*γ* produced by CD8^+^ cells may be able to induce the upregulation of* Gbp1*,* Gbp2,* and* Iigp1* at 3 dpi and then the expression of IFN-*γ* was upregulated through feedback at 10 dpi. As the previous study showed, IFN-*γ*-producing cells were detected as early as 2 dpi in the spleen and the peak IFN-*γ* production by MHC class I-restricted CD8^+^ T cells was presented at 8 dpi [12].

### 3.3. Pathway Analysis of ECTV Infection in Two Mouse Strains at Different Time Points

**Figure 2:**
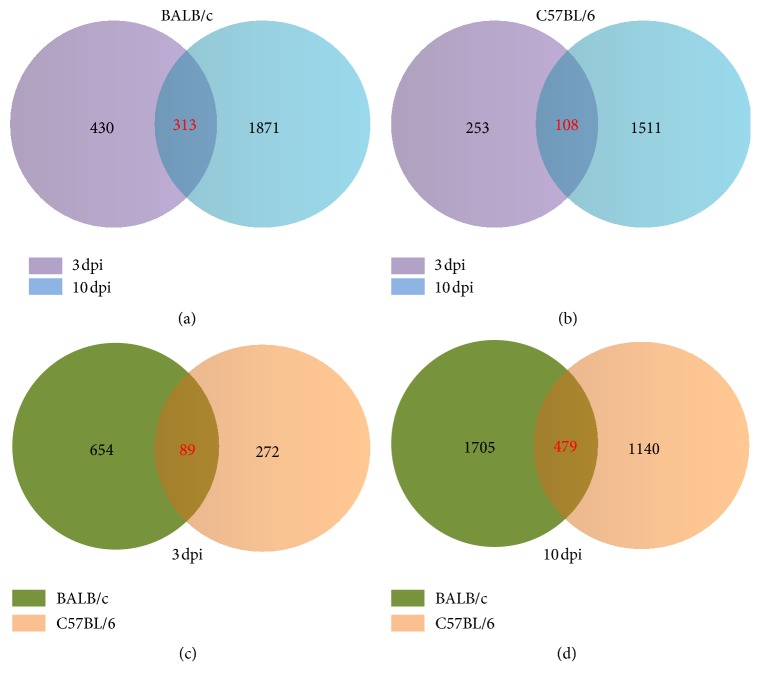
Analysis of common and unique gene expression differentials in two mouse strains at different times of postinfection. Venn diagrams of differentially expressed genes (DEGs) in BALB/c (a) and C57BL/6 (b) mice at 3 days and 10 days after infection (dpi). Venn diagrams of differentially expressed genes (DEGs) at 3 dpi (c) and 10 dpi (d) in two mouse strains.

We constructed Venn diagrams to gain insight into the DEGs that were either unique or shared at the different time points and/or in the different mouse strains. As shown in Figures 2(a) and 2(b), 313 genes in BALB/c mice and 108 genes in C57BL/6 mice were common to all time points. KEGG pathway analysis of the 313 common genes revealed that the most statistically significant (*P *< 0.05) canonical pathways (ranked by* P* value) were the T cell receptor signaling pathway, spliceosomes, antigen processing and presentation, prostate cancer, natural killer cell mediated cytotoxicity, the cytosolic DNA-sensing pathway, the Toll-like receptor (TLR) signaling pathway, and the NOD-like receptor signaling pathway. Only the B cell receptor signaling pathway, leukocyte transendothelial migration, and MAPK signaling pathway were included in the 108 DEGs in C57BL/6 mice. For the cytosolic DNA-sensing pathway and TLR signaling pathway, DEGs including* Zbp1* (Z-DNA binding protein 1),* Cxcl10* (chemokine (C-X-C motif) ligand 10),* Chuk* (conserved helix-loop-helix ubiquitous kinase),* MAP2K4* (mitogen-activated protein kinase kinase 4), and* STAT1* (signal transducer and activator of transcription 1) showed increased expression in the susceptible mice.

The number of unique genes at 3 and 10 dpi in BALB/c mice was 430 and 1871, respectively. Of the 430 unique genes were those that could be classified under canonical pathways including natural killer cell mediated cytotoxicity, Wnt signaling, and allograft rejection. At 10 dpi, the canonical pathways associated with the 1871 unique transcripts included cysteine and methionine metabolism, tyrosine metabolism, cytokine-cytokine receptor interaction, chemokine signaling pathway, complement and coagulation cascades, and Jak-STAT signaling pathway. All of these pathways are important in metabolism and host response, and the majority of genes involved showed increased expression in the susceptible mice.

In the C57BL/6 mice, 253 and 1511 unique transcripts were altered at 3 and 10 dpi, respectively. At the early challenge (3 dpi), fewer unique transcripts were differentially expressed that were involved in Fc gamma R-mediated phagocytosis, lysosome, and leukocyte transendothelial migration. Of the 1511 unique genes differentially expressed at 10 dpi, the most statistically significant canonical pathways were colorectal cancer, intestinal immune network for IgA production, DNA replication, and Fc Epsilon Receptor 1 signaling pathway. The genes involved in these pathways were mainly downregulated in the resistant mice.

Venn diagrams relating the same time points in different mouse strains showed that only 89 and 479 common DEGs were altered at 3 and 10 dpi, respectively (Figures 2(c) and 2(d)). KEGG pathway analysis revealed that no canonical pathways were associated with those 89 common genes at 3 dpi, while the 479 common altered genes at 10 dpi could be classified into canonical pathways that included natural killer cell mediated cytotoxicity and apoptosis. For natural killer cell mediated cytotoxicity,* Fasl* (Fas ligand),* Fcgr4* (Fc receptor, IgG, low affinity IV),* GzmB* (granzyme B),* Ifn-γ* (interferon gamma),* Klrc1* (killer cell lectin-like receptor subfamily C, member 1), and* Klrk1* (killer cell lectin-like receptor subfamily K, member 1) showed increased expression, whereas only* Cd244* (CD244 natural killer cell receptor 2B4) showed decreased expression.

The numbers of genes unique at 3 and 10 dpi time points were 654 and 1705 in BALB/c mice, which were 272 and 1140 in C57BL/6 mice, respectively. At 3 dpi, the 654 unique genes in BALB/c mice were aligned with the canonical pathways associated with ubiquitin mediated-proteolysis, toxoplasmosis, and T cell receptor signaling pathways, while the 272 genes in C57BL/6 mice were only involved in leukocyte transendothelial migration, HIF-1 signaling pathway, and Fc gamma R-mediated phagocytosis. All these pathways were important in regulation of immune response process. At 10 dpi, the pathways associated with 1705 unique transcripts in BALB/c mice encompassed protein digestion and absorption, biosynthesis of amino acids, transcriptional misregulation in cancer, glycerolipid metabolism, and Jak-STAT signaling pathway. Of the 1140 unique genes differentially expressed in C57BL/6 mice, the most statistically significant canonical pathways were HTLV-I infection, protein processing in endoplasmic reticulum, MAPK signaling pathway, intestinal immune network for IgA production, and T cell receptor signaling pathway. A higher proportion of the genes involved in adaptive immune response of C57BL/6 mice suggested a robust antiviral response against ECTV infection.

Taken together, these results suggest that ECTV infection affects the expression of genes involved in molecular and cellular functions. More pathways involved in the host metabolism and innate immune response to infection were induced in the susceptible BALB/c mice, such as metabolism of amino acids and innate immune signaling in nucleic acid recognition. The C57BL/6 mice showed resistance to the infection and more adaptive immune-related pathways were therefore affected during the infection, suggesting the different genetic factors and adaptive immune response are the most important to control the infection.

### 3.4. Differential Expression Levels of Innate Immune Genes in Two Mouse Strains during Infection

The innate immune system represents the first line of host defense against pathogen infection. Various elements of the innate immune response have been implicated in the cellular reaction to, and restriction of, viral infection, including type I and type II IFNs, ISGs, chemokines, interleukins, granzymes, and innate immune cells (including dendritic cells, macrophages, and NK cells). To obtain transcriptomic information about these genes and innate immune cells related genes, we assessed differences in the expression level of selected genes involved in the innate antiviral immune response in the two mice strains during ECTV infection.

We performed a DEGs analysis of ISGs stimulated by ECTV infection in C57BL/6 and BALB/c mice at 3 and 10 dpi (Table 3). The results revealed that more ISGs were upregulated at 10 dpi than at 3 dpi in both C57BL/6 and BALB/c mice. Furthermore, all of these genes were more strongly upregulated at 10 dpi, suggesting a reinforcement of differential gene expression over time. In addition, almost all of these genes were more strongly upregulated in BALB/c mice than in C57BL/6 mice, and some of these genes were upregulated only in BALB/c mice, suggesting they are more sensitive to ECTV infection. Of note, some members of the interferon-induced GTPase family, including* GBP1*,* 2*,* 3*,* 7*, and* 8*, were found to follow a similar upward trend and were more strongly upregulated than other genes in both BALB/c and C57BL/6 mice, suggesting the importance of GBPs in the response to ECTV infection.

Finally, differences in chemokines, interleukins, and granzymes were examined in the two mouse strains (Table 4). IFN-*ζ* and IFN-*α*2 were upregulated in C57BL/6 mice but not in BALB/c mice, and this was true for* GzmA *and* IL1F9 *expression.* GzmB* was strongly upregulated in both mouse strains at the late stage of viral infection, while other members (*GzmC*,* GzmD*,* GzmE*,* and GzmF*) were upregulated only in BALB/c mice at 10 dpi. Some chemokines, such as* Cxcl1*,* Cxcl5*,* Cxcl9*,* Cxcl10*,* Cxcl11,* and* Ccl3,* were upregulated in BALB/c mice at 3 and 10 dpi but were only slightly upregulated in C57BL/6 at 10 dpi. As might be expected, interleukins, complement, and some immunoregulatory molecules were upregulated in BALB/c mice but were only slightly changed, or not at all, in C57BL/6 mice. Members of the killer cell lectin-like receptor subfamily were also affected by ECTV infection.* KLRC1* and* KLRK1* were upregulated in both C57BL/6 and BALB/c mice at 10 dpi, but* KLRG1* was upregulated only in C57BL/6 mice. Taken together, these results suggest that a more enhanced innate immune response to ECTV infection occurred in BALB/c mice than in C57BL/6 mice, which may reflect fundamental differences in the genetic background of the host.

### 3.5. Validation of Microarray Data

**Figure 3:**
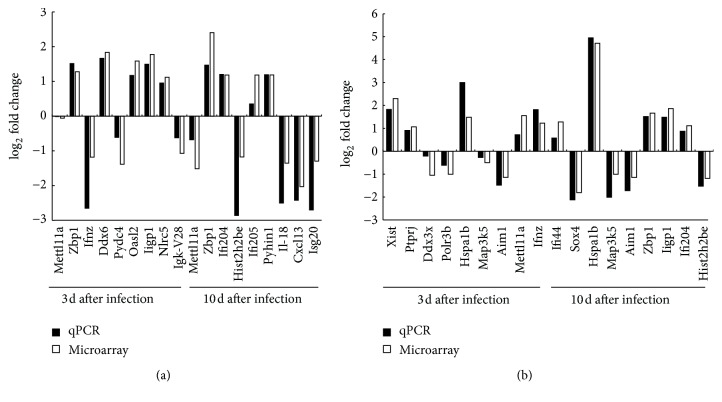
Verification of the gene expression by qRT-PCR. Nine differentially expressed genes (DEGs) containing up- or downregulated genes in microarray analysis at each time point (3 and 10 dpi) were selected randomly for validation of the RNA-seq data. Gene expression in ECTV-infected C57BL/6 (a) and BALB/c (b) mice using qRT-PCR was analyzed using the 2^−ΔΔCt^ method.

To validate the microarray data, we used the same RNA samples to perform qRT-PCR. We measured the expression of 26 upregulated and downregulated genes at each postinfection time point for the two mouse strains. The selected genes are mostly involved in innate immune response which are interested in our future work. As shown in Figure 3, the qRT-PCR results were largely consistent with the microarray analysis. For some genes, however, the fold change values were lower in the qRT-PCR data than in the microarray results. These included* IFNZ* (0.16-fold versus 0.44-fold),* OASL2* (2.25-fold versus 3.01-fold),* ZBP1 *(2.77-fold versus 5.29-fold),* IFI205* (1.27-fold versus 2.27-fold) in BALB/c mice, and* METTL11A* (1.65-fold versus 2.91-fold) and* IFI44* (1.49-fold versus 2.42-fold) in C57BL/6 mice. This discrepancy is likely due to the different detection methods. Overall, these results validate our microarray data and they can therefore be used to infer biological relevance.

## 4. Discussion

ECTV infection can lead to different outcomes in inbred mouse strains. Some strains are susceptible to severe disease and have a high mortality rate, while others, such as C57BL/6, C57BL/10, AKR, and some sublines of 129 mice, show resistance to the virus [12–15]. This is not only due to virus strain, virus immune evasion strategies, dose, and route of infection, but also due to the genetic background of the host [9, 11]. Over the past decades, a number of works have shown that the numerous host factors associated with innate and adaptive immune responses are essential for resistance to mousepox [11]. However, details regarding the host immune response to ECTV infection in genetically susceptible and resistant mice remain to be elucidated. To address this, we used a well-defined mousepox model, with BALB/c as the susceptible strain and C57BL/6 as the resistant strain, which were challenged with ECTV. The susceptible strain reflected a significantly higher virus titer in spleen tissues and one animal death at 10 dpi, but the resistant C57BL/6 strain showed no significant symptoms and no animal deaths. Of note, the virus was detectable in the spleen of BALB/c mice at 3 dpi, but not in C57BL/6 mice, suggesting the importance of genetic background. So far, at least 4 genetic loci in the mouse genome are known to confer resistance to mousepox [16]. The susceptible BALB/c mice were found to be lacking these resistance alleles and the lack of these immunity related genes leads to weak and delayed immune response against ECTV infection [15–18].

Transcriptomic studies provide useful information about underlying pathogenic mechanisms of different genetic backgrounds and interactions following a course of virus infection [41, 42]. In the current study, we utilized microarray technology to examine the host gene expression profiles of susceptible and resistant mice in response to ECTV infection. Our analysis showed that ECTV strongly altered gene expression in both mouse strains. In particular, gene expression was greatly altered at the late stage of infection, and more genes were altered in the susceptible mice than in the resistant mice during the course of the infection. In addition, more upregulated genes than downregulated genes were observed in the BALB/c mice which was the opposite result to that for the C57BL/6 mice. These observations may be the result of a higher viral loads in the spleen of BALB/c mice that would in turn affect the expression of more host genes. We showed that a number of genes were upregulated during infection in the susceptible BALB/c mice, but these were unchanged or downregulated in the resistant C57BL/6 mice, suggesting different mechanisms exist in the two mouse strains in response to ECTV infection. These DEGs could potentially be the key to understanding the different pathologies associated with the two mouse strains. Of note,* Hspa1b* was found to be upregulated in both mouse strains during infection. Previous studies on the transcriptome of host cells during VACV infection also showed* Hspa1b* upregulation, and data from RNAi screens identified a necessary role for Hspa1b in* Orthopoxvirus* infection [43, 44].

We performed pathway analyses of shared DEGs at different time points after ECTV infection in two different mouse strains. Pathways involved in the innate and adaptive immune systems in the control of ECTV infection were found in the susceptible BALB/c mice. These include nucleic acid recognition pathways, natural killer cell mediated cytotoxicity, and the APC-TCR signaling pathway. Nucleic acid recognition pathways are important components of the innate immune system, which serves as the first line of defense and directs subsequent events to activate the host's adaptive immune system [45, 46]. PRRs, including TLR9, STING, and their relevant adaptor Myd88 and nuclear transcription factors, IRF3 and IRF7, are essential for resistance to mousepox [28, 29]. And also, the importance of these molecules in response to the infection has been addressed in VACV, CPXV, and MPXV [30, 41]. Other PRRs, such as cGAS, a critical cytosolic DNA sensor, were speculated that it plays an essential role in inherent resistance to mousepox [28]. In the present study, the expression of these genes was unchanged or slightly upregulated in the two mouse strains which may be due to their expression in certain cell types and/or tissues [47–49]. Despite less genes induced in resistant C57BL/6 mice, the commonly affected genes during the infection were highly enriched in leukocyte transendothelial migration and MAPK signaling pathway, which were also affected by CPXV and MPXV [41].

NK cells are part of the first line of defense to viral infection. The importance of NK cells in defense against poxviruses has emerged over several decades, and they have been shown to play an essential role in inherent resistance to mousepox [50–52]. A number of previous studies have found increased numbers of NK cells in popliteal lymph nodes, spleen, and liver after infection, with peak NK activity occurring at 5 days after infection in both susceptible and resistant mouse strains [52, 53]. Depletion studies have shown that severe infection occurs in resistant C57BL/6 mice, and the NK response is required for resistance during the first few days, so that by day 5 the depletion does not have a major impact on recovery [52, 54]. Our analyses showed that NK cells and NK cell mediated cytotoxicity were stimulated in both mouse strains at 10 dpi, indicating the importance of NK cells [10]. In addition, the delayed induction of NK response presented in our study might be explained by the different routes of infection and the less virulence of the virus strain used in our work. And also, the upregulation of NK cells related genes were observed late in the spleens, where maybe they act earlier than our detection time or are secreted from other tissues. Granzymes (Gzm) are serine proteases expressed by cytotoxic T cells and NK cells and are important for the destruction of virally infected cells [55]. C57BL/6 mice deficient in both GzmA and GzmB are susceptible to mousepox, while moderate susceptibility to the virus is seen in mice that are deficient in only one, demonstrating some overlap between these two effectors [11, 56]. In the present study,* GzmB* was strongly upregulated in the two mouse strains at 10 dpi, suggesting an adaptive immune response and more specifically that cytotoxic T lymphocytes may have taken over the response. Other granzymes were also increased mainly in the BALB/c mice, suggesting a stronger immune response in the susceptible strain.

Type I and II IFNs are among the first cytokines to be produced during viral infection and are essential for inherent resistance to mousepox. Both types of IFN induce the expression of ISGs, which have a variety of functions ranging from direct inhibition of viral components to activation of other immune cell types. C57BL/6 mice deficient in IFN-*α* and IFN-*β* showed increased mortality and enhanced viral loads following ECTV infection [11]. In addition, resistant mice (C57BL/6 and 129) with a targeted deletion of the IFN-*α*/*β* receptor are highly susceptible to mousepox [11, 27]. Results from the current study also showed increased expression of IFN-*ζ* and IFN-*α*2, as well as ISGs, in resistant mice, suggesting that they play an important role in the control of ECTV infection. IFN-*γ* is produced by NK cells and CD8^+^ T cells. The essential role of IFN-*γ* in the control of ECTV infection was confirmed in IFN-*γ*-deficient C57BL/6 mice, which are highly susceptible to mousepox and promote ECTV spread* in vivo *[11, 12]. However, ECTV encodes an IFN-*γ* decoy receptor, which binds directly to the host IFN-*γ* with high affinity and blocks cytokine action extracellularly, prior to receptor engagement [57]. ECTV deficient in this molecular is mildly attenuated, suggesting other ECTV-encoded factors may modify the function of IFN-*γ* [58].

In summary, we characterized global gene expression patterns that are shared and distinct between the spleen tissues from ECTV-susceptible and ECTV-resistant mouse strains. The susceptible mice showed a stronger response to the infection with higher viral loads than the resistant strain. Our results highlight differences in the response to ECTV between ECTV-susceptible and ECTV-resistant mice. Although a global overview of some events occurring in ECTV-susceptible and ECTV-resistant mice was observed by using microarray analysis, the complicated mechanisms of host responses in different mouse strains were not clearly elucidated. Therefore, considering the data in the present study, more detection time points and target tissues, such as skin, blood, liver, and regional lymph nodes, should be performed in the future.

## Figures and Tables

**Table 1:** Total number of differentially expressed genes (DEGs)^a^.

Mouse strain	BALB/c		C57BL/6	
After infection	3 dpi	10 dpi	3 dpi	10 dpi
Number of upregulated genes	470	1453	123	540
Number of downregulated genes	273	731	238	1079
Total number	743	2184	361	1619

^a^Microarray data of infected groups were normalized with uninfected group of each mouse strain. The DEGs were filtered using the criterion of cutoff limitation as a fold change ≥ 2 or ≤0.5.

**Table 2:** List of 10 DEGs that were most up- or downregulated in BALB/c and C57BL/6 during ECTV infection^b^.

Mouse strain	BALB/c				C57BL/6			
After infection	3 dpi versus uninfected		10 dpi versus uninfected		3 dpi versus uninfected		10 dpi versus uninfected	
Gene category	Gene	Fold change	Gene	Fold change	Gene	Fold change	Gene	Fold change
Upregulated	*Kap*	5.65	*Spp1*	46.2	*Xist*	27.28	*Xist*	50.37
	*Hspa1b*	4.39	*Gzmb*	39.4	*Ttr*	4.49	*Hspa1b*	26.23
	*Gbp1*	4.32	*Ifng*	24.2	*Tsks*	3.13	*Gzmb*	22.26
	*Gdi2*	3.99	*Vcan*	20.7	*Mettl11a*	2.93	*Gbp1*	17.84
	*Apol7c*	3.95	*Mmp3*	17.9	*Hspa1b*	2.81	*Hspa1a*	15.25
	*Slfn4*	3.60	*Nts*	16.5	*Ptger1*	2.68	*Ctsg*	14.02
	*Gbp2*	3.57	*Timp1*	14.0	*Alb*	2.59	*Mcpt8*	10.06
	*Ddx6*	3.47	*Gzmd*	13.6	*Ifnz*	2.34	*Rgs1*	8.74
	*Iigp1*	3.42	*Saa3*	13.6	*Dppa5a*	2.32	*Gzmk*	7.71
	*Acaa2*	3.41	*Cxcl5*	12.6	*Cml3*	2.29	*Prtn3*	7.03

Downregulated	*Vmn1r148*	0.33	*Ctrb1*	0.08	*Psap*	0.29	*Bpgm*	0.17
	*Phxr5*	0.33	*Try4*	0.02	*Cyp4f16*	0.28	*Dbp*	0.17
	*Reg2*	0.32	*Cpb1*	0.03	*Foxp1*	0.28	*Igfbp5*	0.17
	*Psg28*	0.29	*Cela3b*	0.03	*Epsti1*	0.28	*Ccr9*	0.16
	*Mup10*	0.28	*Marco*	0.03	*Abhd12*	0.27	*Apol7c*	0.16
	*Astx*	0.25	*Clps*	0.04	*Kap*	0.27	*Kdm5d*	0.16
	*Adipoq*	0.25	*Zg16*	0.05	*Tpm3*	0.26	*Cyr61*	0.15
	*Cfd*	0.16	*Emr4*	0.06	*Ddx3y*	0.25	*Ddx3y*	0.14
	*Car3*	0.08	*Pnlip*	0.02	*Sh3bgrl*	0.25	*Eif2s3y*	0.13
	*Mettl11a*	0.06	*Amy2a1*	0.006	*Hmgcs1*	0.22	*Igfbp3*	0.12

^b^The DEGs were ranked by fold change and those were the most (fold change) up- or downregulated in expression at 3 and 10 dpi in BALB/c and C57BL/6 mice.

**Table 3:** The interferon-stimulated genes (ISGs) that were upregulated in BALB/c and C57BL/6 during ECTV infection^c^.

Genes			3 dpi^*∗*^		10 dpi^*∗*^	
Gene symbol	RefSeq	Gene name	B^i^/B^u^	C^i^/C^u^	B^i^/B^u^	C^i^/C^u^
OASL1	AB067533	2′-5′-Oligoadenylate synthetase-like 1	1.91	0.82	3.34	1.21
OAS2	AB067535	2′-5′-Oligoadenylate synthetase 2	1.90	1.13	4.15	1.23
IGTP	NM_018738	Interferon gamma induced GTPase	2.03	0.88	2.48	2.39
IFI47	NM_008330	Interferon gamma inducible protein 47	2.07	0.81	2.60	1.66
IFIT2	NM_008332	Interferon-induced protein with tetratricopeptide repeats 2	1.54	0.80	3.69	1.29
IIGP1	BM239828	Interferon inducible GTPase 1	3.42	0.96	5.32	3.64
IFI204	NM_008329	Interferon activated gene 204	1.52	0.85	6.10	2.16
IFI202B	NM_011940	Interferon activated gene 202B	1.49	0.90	4.00	1.15
IFI44	BB329808	Interferon-induced protein 44	2.49	0.89	4.93	2.42
IFITM1	BC027285	Interferon induced transmembrane protein 1	1.03	0.95	2.10	1.36
IFITM3	BC010291	Interferon induced transmembrane protein 3	1.09	1.13	1.73	1.49
IFITM6	BB193024	Interferon induced transmembrane protein 6	1.18	1.15	1.92	1.86
IFIH1	AY075132	Interferon induced with helicase C domain 1	1.57	1.17	1.82	1.42
ISG15	AK019325	Interferon-stimulated protein (15 kda)	1.73	1.22	1.93	0.92
GBP1	NM_010259	Guanylate binding protein 1	4.32	0.86	6.60	17.84
GBP2	NM_010260	Guanylate binding protein 2	3.60	0.82	7.15	3.28
GBP3	NM_018734	Guanylate binding protein 3	1.82	0.98	3.27	2.l0
GBP7	BC010229	Guanylate binding protein 7	2.39	1.04	3.61	2.44
GBP8	NM_029509	Guanylate binding protein 8	1.69	1.21	2.24	3.20
MX1	M21039	Myxovirus resistance 1	1.29	1.16	3.30	1.05
EIF2*α*K2	AV328340	Eukaryotic translation initiation factor 2-*α* kinase 2	2.17	0.97	2.49	0.91
CH25H	NM_009890	Cholesterol 25-hydroxylase	0.97	0.84	2.68	0.84

^c^The interferon-stimulated genes (ISGs) differentially expressed in BALB/c and/or C57BL/6 were selected for analysis at 3 and 10 dpi. ^*∗*^The capital “B” and “C” represent BALB/c and C57BL/6 mice, respectively. Superscripts “i” and “u” represent infected and uninfected mice, respectively.

**Table 4:** The cytokines that were upregulated in BALB/c and C57BL/6 during ECTV infection^d^.

Genes			3 dpi^*∗*^		10 dpi^*∗*^	
Gene symbol	RefSeq	Gene name	B^i^/B^u^	C^i^/C^u^	B^i^/B^u^	C^i^/C^u^
IFN-*γ*	K00083	Interferon gamma	0.95	0.89	24.17	5.21
IFN-*ζ*	BF022827	Interferon zeta	0.44	2.34	0.89	1.00
IFN-*α*2	NM_010503	Interferon alpha 2	0.79	1.76	0.82	1.54
GzmA	NM_010370	Granzyme A	0.68	1.83	1.13	5.51
GzmB	NM_013542	Granzyme B	1.12	0.87	39.39	22.26
GzmC	NM_010371	Granzyme C	0.92	0.89	5.96	0.96
GzmD	NM_010372	Granzyme D	1.07	0.95	13.63	1.02
GzmE	NM_010373	Granzyme E	1.33	0.98	10.21	1.07
GzmF	NM_010374	Granzyme F	0.88	0.99	2.68	1.22
GzmK	AB032200	Granzyme K	0.61	0.93	7.52	7.71
KLRG1	NM_016970	Killer cell lectin-like receptor subfamily G, member 1	0.67	1.17	1.06	3.56
KLRC1	AF106008	Killer cell lectin-like receptor subfamily C, member 1	1.19	0.67	5.57	1.89
KLRK1	AF039026	Killer cell lectin-like receptor subfamily K, member 1	1.13	1.13	2.72	2.15
CXCL1	NM_008176	Chemokine (C-X-C motif) ligand 1	2.43	1.16	7.16	1.99
CXCL5	NM_009141	Chemokine (C-X-C motif) ligand 5	2.83	0.90	12.61	1.86
CXCL9	NM_008599	Chemokine (C-X-C motif) ligand 9	1.45	1.07	3.99	2.86
CXCL10	NM_021274	Chemokine (C-X-C motif) ligand 10	2.41	0.93	5.46	2.21
CXCL11	NM_019494	Chemokine (C-X-C motif) ligand 11	1.47	1.11	5.19	1.07
CCL2	AF065933	Chemokine (C-C motif) ligand 2	1.14	1.11	5.86	1.15
CCL3	NM_011337	Chemokine (C-C motif) ligand 3	1.73	0.97	12.46	4.52
CCL4	AF128218	Chemokine (C-C motif) ligand 4	0.85	1.23	4.65	2.72
CCL6	AV084904	Chemokine (C-C motif) ligand 6	1.68	0.73	3.04	0.90
CCL7	AF128193	Chemokine (C-C motif) ligand 7	0.98	0.95	3.34	1.79
CCL8	NM_021443	Chemokine (C-C motif) ligand 8	0.85	1.26	4.16	4.29
CCL12	U50712	Chemokine (C-C motif) ligand 12	1.08	1.35	4.81	1.41
IL1-*α*	BC003727	Interleukin 1 alpha	1.06	1.08	1.33	1.74
IL1R2	NM_010555	Interleukin 1 receptor, type II	1.45	0.89	8.99	1.99
IL1F9	AY071843	Interleukin 1 family, member 9	1.23	1.90	0.77	1.71
IL1RAP	NM_134103	Interleukin 1 receptor accessory protein	1.30	1.17	1.95	1.65
IL1RN	M57525	Interleukin 1 receptor antagonist	1.17	0.75	2.73	1.06
IL2R-*β*	M28052	Interleukin 2 receptor, *β* chain	1.11	0.56	2.64	0.95
IL2R-*α*	AF054581	Interleukin 2 receptor, *α* chain	1.03	0.70	5.48	0.54
IL12R-*β*1	NM_008353	Interleukin 12 receptor, *β*1	1.15	0.97	2.65	1.59
IL33	NM_133775	Interleukin 33	1.05	0.84	3.83	0.81
STAT1	BB229853	Signal transducer and activator of transcription 1	2.97	0.80	6.34	1.11
STAT2	AF088862	Signal transducer and activator of transcription 2	1.77	1.01	2.04	1.60
STAT3	BG069527	Signal transducer and activator of transcription 3	1.50	1.38	2.73	0.93
TNFRSF9	BC028507	Tumor necrosis factor receptor superfamily, member 9	1.03	0.97	6.23	1.06

^d^The cytokines differentially expressed in BALB/c and/or C57BL/6 were selected for analysis at 3 and 10 dpi. ^*∗*^The capital “B” and “C” represent BALB/c and C57BL/6 mice, respectively. Superscripts “i” and “u” represent infected and uninfected mice, respectively.

## Supplementary Materials

Supplementary 1 Table S1: genes and primers used in qRT-PCR validation.

Supplementary 2 Table S2: the list of the differentially expressed genes in BALB/c and C57BL/6 mice infected with ECTV at 3 and 10 dpi.
