# Supplementary material for: Comparison of Host Gene Expression Profiles in Spleen Tissues of Genetically Susceptible and Resistant Mice during ECTV Infection
Source: Biomed Res Int. 2017 Dec 21;2017:6456180. doi: 10.1155/2017/6456180 (PMC5752998; doi:10.1155/2017/6456180)
Supplement: Supplementary 1 — Table S1: genes and primers used in qRT-PCR validation. [file 6456180.f1.pdf]

Supplementary Materials: Table S1. Genes and primers used in qRT-PCR validation.

Gene name	Accession No.	Primer sequence (5′ to 3′)	Product length (bp)
Gapdh	AI840508	GGTTGTCTCCTGCGACTTCA	183
		TGGTCCAGGGTTTCTTACTCC	
Xist	L04961	CTGCGTGATACGGCTATTCTC	196
		CACTCCTTGGCTTTCTACTTCC	
Ptprj	D83204	TGGCAGAATACGGACGATG	194
		TACTGGGTAATGGAGTTGGAGAT	
Ddx3x	L25126	ACTCCCACCAAGTGAACGATT	192
		GGGCGAGTATAACGAGTAAGCT	
Polr3b	C85372	TTATGGCAACAAACGACTGGA	122
		CTCTCTGTTTAGGAATCACTTGGTC	
Ifi44	BB329808	AAACCATCCGAACTATACCCAT	193
		CAGATCCAGGCTATCCACGT	
Sox4	BG083485	GGGGAAAAGAAAAGGAAGAAA	187
		CCACCACCAACATCAATAACAA	
Hspa1b	M12573	TTCAAAGTAATGTTGGGAGCAG	148
		GAACTGGGCAGCTAGACTATATGT	
Map3k5	NM_008580	ACCAGCAGCAGTAGCGAGTAT	113
		ATCCCTTTCCCGTCCTTTT	
Aim1	BM233292	GAAGTCAAACCTGCCGAATG	223
		GATGTCCCCAAACACCTCAAT	
Ddx3y	AA210261	CCGTAAACAATACCCAATCTCC	225
		CCAATCTTTCCTCTTTCCATCA	
Mettl11a	AV309800	TGACGAGCGAGGTGATTGA	188
		GGAAGTCCCTGTCTTGTTTGG	
Zbp1	AK008179	GAATGACGACAGCCAAAGAAG	162
		GACAAATAATCGCAGGGGACT	
Ifnz	BF022827	TTCCGAGGTTTCTTCCGCT	201
		CCGTGCGTCTGCTTTGAAT	
Iigp1	NM_021792	TCCACATTAGGTAGAGCAAGGG	156
		TCAATGCTAACCCTGAACACG	
Ifi204	NM_008329	TGGAGCAGTGTTCTATGGAGTGT	206
		CACACACTAACTTTGGTTGCCT	
Hist2h2be	AV127319	AACTGAAACCGCTCTGTCCCT	125
		TACCAAATCGGCAGCAACG	
Ddx6	BF226295	ATCAGGTCGCTTTGGTCATC	190
		GCGTGCTTGTTACGGTTTCT	
Nlrc5	AV277444	AGGAGTTTGCCTACTATGGGG	216
		TCGGCTCAGGTCAAGTTTCT	
Igk-V28	AY058908	CCACCATCCAGTGAGCAGTTA	130

		TGTTCAGGACGCCATTTTGT	
Pydc4	BB135602	TCCAGAACTTGCAGCTCGTGT	111
		ATCAGGCTGGAGGTAAGTGGAG	
Ifi205	AI481797	CAGCCCAGAAAAGGAAAGGT	141
		CCGAAGATGAGACCTGGGATA	
Pyhin1	BM241008	AGATGTTCCATGCCACCGT	185
		CATTGGTTCGCCATCATTTAC	
Il18	NM_008360	GGACACTTTCTTGCTTGCCA	209
		CAGCCTCGGGTATTCTGTTATG	
Cxcl13	AF030636	GCATACCCAACCCACATCCT	197
		AAGTCCATCTCGCAAACCTCT	
Isg20	BC022751	ACTGCTCTGTGGAAGATGCC	125
		TCTAACCCTGGATGAGGATGAA	
Oasl2	BQ033138	ACCCTAAAGGTTCAGTCCCG	125
		ATCAGAGTCTCGTAGATTGCCG	
